# ADHD and autism in Neurocognitive Mismatch Theory: distinct neurodevelopmental incompatibilities with the market-based system

**DOI:** 10.3389/fpsyg.2025.1617192

**Published:** 2025-08-05

**Authors:** Joseph Lewis Kidwell

**Affiliations:** Independent Researcher, Willmar, MN, United States

**Keywords:** ADHD, autism, neurodevelopment, neurodiversity, market-based systems, neuroplasticity, neurocognitive mismatch

## Abstract

ADHD and Autism Spectrum Disorder (ASD) represent distinct neurodevelopmental conditions with unique profiles, yet they share susceptibility to environmental pressures that may exacerbate cognitive mismatches. This paper argues that Attention-Deficit/Hyperactivity Disorder and Autism Spectrum traits are not fixed neurological disorders but neurodevelopmental variants destabilized by the sociobiological mismatch between evolved human cognition and the pressures of modern market-based civilization. Drawing on evolutionary biology, developmental neuroscience, social epidemiology, and political economy, the paper reframes these conditions as context-contingent outcomes: traits that are biologically conserved due to their adaptive value in ancestral environments but rendered dysfunctional under chronic stress, inequality, overstimulation, environmental toxicity, and cognitive suppression endemic to industrial societies. It synthesizes evidence across prenatal programming, intergenerational stress transmission, pharmaceutical ethics, and neuroplastic adaptation to propose an ecological model in which the environment, not the brain, is the primary source of pathology. This reframing calls for systemic transformation, not individual correction, and provides a foundation for more inclusive, developmentally respectful, and ecologically coherent mental health paradigms.

## 1 Introduction: from pathology to context

It is important to emphasize that while ADHD and ASD are discussed in parallel, they possess distinct neurological and developmental trajectories. ADHD is typically defined as a neurodevelopmental disorder marked by inattention, impulsivity, and hyperactivity. Autism spectrum conditions are described as developmental impairments in social communication, flexibility, and sensory processing. Both are commonly treated as intrinsic, biologically fixed disorders, requiring therapeutic intervention or pharmaceutical regulation. This paper rejects that framing. It argues that what we label as disorder is in fact a sociobiological mismatch: an emergent dysfunction caused not by the individual’s brain but by its forced adaptation to an artificial, misaligned environment (Arnsten, 2009; Walker, 2021). By recentering analysis on context, this framework does not deny biology, it reinterprets it. ADHD and autism spectrum traits are biologically real and neurodevelopmentally rooted. But their dysfunction is not predetermined. It arises when cognitive systems, designed for adaptive functioning in mobile, communal, and dynamic ancestral environments, are forced into rigid, overstimulating, sedentary, and hierarchically constrained systems (Crespi, 2016; Sapolsky, 2005). The result is not a disease but a reaction, a neurocognitive incompatibility with the structure of market civilization.

## 2 Theoretical foundations and prior models

This section meticulously positions the Neurocognitive Mismatch Theory (NMT) within the broader landscape of existing theoretical models concerning neurodevelopmental conditions. It articulates how NMT builds upon, aligns with, extends, or contrasts with established paradigms, thereby demonstrating a sophisticated understanding of the intellectual lineage while clearly delineating NMT’s unique contributions.

### 2.1 Clinical definitions and diagnostic frameworks

According to the DSM-5-TR (American Psychiatric Association, 2022), Attention-Deficit/Hyperactivity Disorder is characterized by persistent patterns of inattention and/or hyperactivity-impulsivity that interfere with functioning or development. The diagnostic criteria require symptoms to be present before age 12, occur in multiple settings, and cause clinically significant impairment. Similarly, Autism Spectrum Disorder is defined by persistent deficits in social communication and social interaction across multiple contexts, accompanied by restricted, repetitive patterns of behavior, interests, or activities (American Psychiatric Association, 2022). While this paper challenges the deficit-based framing of these definitions, acknowledging their clinical conceptualization is essential for understanding how the Neurocognitive Mismatch Theory departs from mainstream psychiatric models. The DSM-5-TR’s categorical approach assumes these conditions represent discrete pathological entities, whereas our framework proposes they represent contextually-destabilized variations within normal human neurodiversity.

### 2.2 Existing theoretical models

NMT’s relationship with existing theoretical models is carefully articulated, demonstrating a strategic intellectual positioning that integrates prior scholarship while advancing a novel perspective.

#### 2.2.1 Neurodiversity paradigm

The neurodiversity movement, pioneered by advocates like Singer (1998) and elaborated by Walker (2021), conceptualizes neurological differences as natural variations rather than pathologies. Our framework builds upon this foundation but extends it by proposing specific mechanisms through which environmental mismatch converts neutral variation into functional impairment.

#### 2.2.2 Differential susceptibility theory

Belsky and Pluess (2009) proposed that individuals vary in their susceptibility to environmental influences, with some being more affected by both positive and negative experiences. This theory aligns with our model’s emphasis on environmental sensitivity but differs in that we specifically examine how modern societal structures systematically disadvantage certain neurodevelopmental profiles.

#### 2.2.3 Evolutionary mismatch hypothesis

Gluckman and Hanson (2006) introduced the concept of evolutionary mismatch in health contexts, arguing that many modern diseases result from discordance between our evolved biology and contemporary environments. While they focused primarily on metabolic disorders, we extend this framework to neurodevelopmental conditions.

#### 2.2.4 Social model of disability

The social model (Oliver, 1990) distinguishes between impairment (biological difference) and disability (societal barriers). Our theory incorporates this distinction but goes further by examining how modern environments actively destabilize neurodevelopmental traits through specific biological mechanisms.

### 2.3 Gaps in existing literature

Current models inadequately address several critical aspects of neurodevelopmental conditions. These include the specific mechanisms through which societal structures convert neurodevelopmental differences into dysfunction, the role of market-based economic systems in shaping cognitive demands, the intergenerational transmission of environmental stress effects on neurodevelopment, and the distinction between inherent neurological variation and environmentally-induced destabilization. The Neurocognitive Mismatch Theory addresses these gaps by integrating insights from political economy, environmental neuroscience, and epigenetics to propose a comprehensive framework for understanding how modern societies create dysfunction from diversity.

### 2.4 Distinct applications to ADHD and autism

While both conditions involve environmental mismatch, the specific mechanisms differ considerably. For ADHD-environment interactions, dopaminergic systems that evolved for immediate reward environments clash with modern delayed gratification demands. Similarly, hypervigilance and rapid attention shifting, which were adaptive for threat detection in ancestral contexts, become liabilities in contemporary sustained attention tasks, while fundamental movement needs conflict directly with sedentary institutional requirements. In contrast, autism-environment interactions manifest differently. Heightened sensory processing, potentially adaptive for environmental monitoring in less complex settings, becomes overwhelming in overstimulating modern contexts. Detail-focused cognition, while valuable for certain tasks, clashes with contemporary demands for rapid task-switching, and social communication differences are amplified by increasingly complex and indirect social structures. These distinct pathways converge in their incompatibility with standardized institutional expectations, but manifest through different neurobiological systems.

## 3 Evolutionary origins of cognitive diversity

Human evolution produced a spectrum of attentional, perceptual, and behavioral traits suited to diverse environmental pressures. ADHD-like traits, novelty seeking, risk tolerance, rapid attentional shifts, were likely advantageous in nomadic, foraging, or sentinel roles. Autism spectrum traits, intense focus, pattern fixation, sensory sensitivity, may have aided precision tasks, environmental monitoring, and information retention (Hartmann, 2019; Walker, 2021). These traits persist not because they are errors but because they were selected. They are part of a cognitive ecology shaped by natural selection. Diversity in cognition was adaptive at the group level. The survival of early human bands depended on multiple strategies: explorers and systemizers, scanners and detailers, social connectors and solitary pattern-makers. What we now call ADHD or ASD may have once been role-specific advantages.

## 4 The market system as a misaligned environment

The post-agricultural, market-based civilization that emerged over the past 10,000 years, especially its industrial and digital phases, represents a radical break from the environments in which human cognition evolved. It imposes expectations that are structurally misaligned with evolved neurodevelopmental traits (Illich, 1976; Foucault, 1977). Market systems reward delay of gratification, uninterrupted focus on abstract tasks, social compliance, and routine repetition. Schools, workplaces, and bureaucracies are optimized for these traits. Those who diverge, by seeking novelty, resisting routine, or responding strongly to sensory input, are framed as defective. ADHD and ASD are not disorders in themselves; they are cognitive profiles rendered dysfunctional by systems that enforce conformity to narrow productivity norms.

## 5 Environmental stress and neurodevelopmental destabilization

Modern market environments are not only misaligned, they are biologically hostile. Chronic stress, social inequality, processed diets, environmental toxins, and information saturation create conditions that actively destabilize cognitive development (Luby et al., 2013; Lupien et al., 2009). Studies in social epidemiology have shown that poverty and inequality induce high allostatic load, disrupt cortisol regulation, and impair neurodevelopment in children (Evans and Kim, 2007; Adler et al., 1994). Even perceived poverty or low social status can trigger chronic stress responses. These stressors disproportionately impact individuals with predisposing neurodevelopmental profiles, leading to more severe functional impairments (Corbett et al., 2016), not because the traits are inherently worse, but because the environment compounds their instability. Research also implicates dietary and environmental toxins such as pesticides, lead, phthalates, and air pollution, in worsening executive function and attention regulation (Bouchard et al., 2010; Perera et al., 2019). ADHD and ASD are not caused by these factors alone, but their severity and visibility are heightened in ecologies of chronic inflammation and neural overstimulation. Brain scans of children exposed to poverty, stress, or malnutrition reveal reduced gray matter and altered connectivity; not signs of defective design, but of adaptive systems damaged by hostile inputs.

## 6 The medicalization of contextual dysfunction

Mainstream psychiatry continues to interpret ADHD and ASD through a deficit-based medical model. While this model recognizes neurodevelopmental differences, it frames them as pathological deviations from normative functioning. It focuses on symptom suppression, via behavioral therapy or pharmaceutical intervention, rather than on contextual redesign (Whitaker, 2010). This model is not neutral. It aligns with institutional and economic incentives. The education system benefits from sedating “disruptive” students. Employers benefit from conformity. Pharmaceutical industries profit from long-term symptom management. Diagnostic labels thus become tools of social discipline; means of enforcing compliance with an environment structured for neurotypical performance. The brain imaging and genetic data used to justify disorder classifications show difference, not defect. Heritability and neurological divergence are not evidence of pathology; they are evidence of variation (Cortese et al., 2015). Many traits with high heritability, such as height or temperament, are not classified as disorders. The pathology lies not in the brain, but in the assumption that difference is dysfunction.

## 7 Prenatal stress and the intergenerational transmission of instability

Cognitive outcomes are not merely shaped by the postnatal environment; they begin in utero and even prior to conception. Maternal stress during pregnancy has been consistently linked to altered fetal brain development. Elevated maternal cortisol, immune activation, and inflammatory signaling during gestation can impact fetal neural architecture, especially in regions involved in emotion regulation, sensory processing, and attention (Luby et al., 2013; Arnsten, 2009). These changes do not result from genetic mutation but from environmental programming. The developing brain adapts to signals of environmental danger. In a chronically stressed mother, the fetus receives biochemical cues that may recalibrate neurological development toward heightened vigilance, impulsivity, or sensory reactivity; traits that can later manifest as ADHD or ASD, particularly in unsupportive environments. Recent research also reveals that paternal contributions matter. Animal studies have shown that chronic stress in male rodents alters the epigenetic content of their sperm; modifying DNA methylation and microRNA profiles in ways that influence offspring stress reactivity and brain development (Rodgers et al., 2013; Bohacek and Mansuy, 2015). Human studies suggest similar effects: men exposed to trauma or adversity may pass epigenetic marks to offspring, increasing the risk of neurodevelopmental challenges (Soubry et al., 2016).

## 8 Industrial acceleration and digital stress: epigenetic and cognitive impacts

This section addresses two distinct forms of stress: social stress, arising from societal hierarchies and inequalities, and digital stress, originating from excessive screen time and sensory overstimulation. While prenatal and intergenerational stress shape neurodevelopmental trajectories before birth, the postnatal environment, especially in modern industrialized and digital societies, further contributes to the destabilization of ADHD and autism traits. The Industrial Revolution marked a shift toward urbanization, hierarchical labor systems, and time-discipline regimes that introduced widespread psychosocial stressors. This trend has intensified in the digital era, with attention-fragmenting technologies, sensory overstimulation, and screen-mediated social disconnection compounding the cognitive demands placed on developing minds (Christakis et al., 2004). Chronic psychological stress has well-documented neurodevelopmental consequences, particularly during sensitive developmental periods (Lupien et al., 2009). Elevated allostatic load disrupts regulatory systems in the prefrontal cortex and hippocampus; regions associated with attention, memory, and executive control (McEwen, 2004). These effects are exacerbated by rising income inequality and sociocultural fragmentation in high-income societies (Sapolsky, 2005). Critically, psychological stress also produces epigenetic alterations that may affect the expression of genes associated with ADHD and autism. It is critical to clarify that these epigenetic effects are environmentally induced and potentially reversible, reflecting the dynamic interplay between genes and the environment (Bohacek and Mansuy, 2015; Soubry et al., 2016; Skinner, 2014). Rodent studies demonstrate that paternal stress can modify sperm microRNA and DNA methylation in ways that affect offspring neuroendocrine regulation (Rodgers et al., 2013; Bohacek and Mansuy, 2015). In humans, similar stress-linked epigenetic changes have been observed at imprinted genes relevant to development and behavior (Soubry et al., 2016). Though direct causation is difficult to establish, these findings support the hypothesis that rising psychosocial stress in post-industrial and digital societies may act as a transgenerational amplifier of neurocognitive mismatch effects.

## 9 ADHD and ASD: distinct profiles, shared structural pressure

ADHD and ASD are distinct neurodevelopmental trajectories. ADHD is associated with dopaminergic regulation and frontal-striatal circuitry. ASD involves connectivity differences, sensory modulation, and social-cognitive variation (Nigg et al., 2012; Modabbernia et al., 2017). What unites them is structural, not neurological. Both profiles are destabilized by ecological pressures: stress, overstimulation, rigidity, and exclusion. Both are punished by systems designed for behavioral standardization. Both are framed as disorders because they fail to conform to institutional norms, not because they fail to function in any absolute sense.

## 10 An ecological model of mental diversity

Mental health frameworks must adopt an ecological perspective. Neurodevelopmental traits should be understood in context, as differentially adaptive depending on environment. Instead of forcing individuals to adapt to harmful systems, we must redesign systems to accommodate cognitive diversity (Walker, 2021; Whitaker, 2010). This means restructuring education to allow for movement, novelty, and non-linear learning. It means restructuring workplaces to value deep focus, creative bursts, and non-conventional problem-solving. It means regulating environmental toxins, reducing inequality, and minimizing overstimulation. It means treating neurodevelopmental outcomes as ecological responses, not personal failures.

## 11 Causal mechanisms and methodological considerations

While this paper synthesizes substantial correlational evidence linking environmental factors to neurodevelopmental outcomes, establishing causation requires careful consideration. The relationship between environmental stressors and ADHD/autism traits likely involves bidirectional causality and complex feedback loops. Several proposed causal pathways merit consideration. Direct biological mechanisms include the cascade from chronic stress to HPA axis dysregulation, which in turn can be associated with altered prefrontal cortex development and ultimately executive function impairments (Lupien et al., 2009). Similarly, environmental toxins may trigger neuroinflammation, potentially altering synaptic connections (Matelski and Van de Water, 2016), while prenatal stress can produce neurodevelopmental variations (Buss et al., 2012). Indirect social mechanisms operate through different pathways. Economic inequality creates resource scarcity that may compromise caregiving quality and contribute to emotional regulation difficulties (Noble et al., 2015). Digital media exposure has been associated with attention problems (Swing et al., 2010), while social interaction difficulties are a core feature of autism (Belmonte et al., 2004). Gene–environment interactions add another layer of complexity. Genetic variants may influence an individual’s responses to environmental inputs (Bakermans-Kranenburg and van IJzendoorn, 2011), and environmental factors may activate or suppress genetic predispositions through epigenetic mechanisms (Kubota et al., 2012). Several methodological challenges complicate this research. Reverse causality presents a significant issue, as children with ADHD or autism traits may evoke different environmental responses, creating apparent environmental “causes.” Confounding variables such as socioeconomic status, parental mental health, and genetic factors often co-occur, making it difficult to isolate specific effects. Measurement limitations arise because most studies rely on retrospective reports or cross-sectional designs, while ethical constraints make experimental manipulation of adverse environments impossible in human studies. To address these challenges, future research should employ longitudinal cohort studies tracking environmental exposures and neurodevelopmental outcomes, natural experiments exploiting policy changes or environmental variations, sibling comparison studies controlling for shared genetic and environmental factors, and animal models examining specific causal mechanisms under controlled conditions.

## 12 Neuroplasticity and the ethics of pharmacological intervention

Neuroplasticity offers a path to adaptivity without pharmacological dependence. Evidence shows that individuals with ADHD can improve regulation and focus through cognitive training, mindfulness, physical activity, and sensory-friendly environments (Cortese et al., 2015). By contrast, stimulant medication alters brain chemistry to increase dopamine and norepinephrine. Long-term use may cause receptor downregulation, dependency, and altered baseline attention (Volkow et al., 2009). Some individuals experience worsened symptoms when stopping medication, not because of the condition itself, but due to neuroadaptive shifts induced by the drugs. This does not mean medication is never appropriate. But its use should follow, not precede, environmental and behavioral supports. Prioritizing neuroplastic strategies honors the brain’s adaptability and avoids converting short-term fixes into lifelong dependencies.

## 13 Cross-cultural perspectives and global applications

The Neurocognitive Mismatch Theory, while primarily drawing from research conducted in Western, industrialized societies, possesses significant applicability across diverse cultural contexts. The mismatch hypothesis predicts that neurodevelopmental destabilization should correlate with specific environmental features and societal demands rather than being inherently tied to geographic location or ethnicity. This section explores how neurodevelopmental traits are perceived and manifest in non-Western settings, the global impact of urbanization, the role of cultural protective factors, and the implications for global mental health initiatives.

### 13.1 Beyond western contexts

Research among indigenous and traditional communities provides compelling evidence for this hypothesis. Studies of autism prevalence in isolated communities like the Amish have reported lower rates, though this may reflect diagnostic practices rather than true prevalence (Grace et al., 2022). In East Asian societies, different cultural values around attention, social communication, and behavioral regulation create distinct manifestations of these traits. South Korean studies, for example, have indicated higher ADHD diagnosis rates (Seo et al., 2022). Japanese research on hikikomori (social withdrawal) suggests cultural-specific expressions of autism-like traits (Kato et al., 2019). Limited research from sub-Saharan Africa reveals analogous patterns. Lower reported ADHD prevalence in rural versus urban areas (Bakare, 2012) has been observed. Furthermore, some cultural interpretations of autism traits in Africa exist (De Vries, 2016).

### 13.2 Urbanization as a global risk factor

Across cultures, urbanization consistently correlates with increased neurodevelopmental diagnoses. Studies have indicated that autism prevalence is higher in urban areas compared to rural areas, with prevalence rate ratios ranging from 2.24:1 to 2.72:1 (Gulsrud et al., 2019). This pattern is also observed in countries like Bangladesh, where prevalence was higher in urban (25/10,000) than rural (14/10,000) areas (Hossain et al., 2017). Urban environments share features that may destabilize neurodevelopment, including pollution, social isolation, overstimulation, and reduced nature contact.

### 13.3 Cultural protective factors

Some cultural practices may buffer against neurodevelopmental destabilization. Extended family systems providing distributed caregiving create multiple attachment relationships and reduce caregiver stress. Integrated physical activity in daily life naturally accommodates movement needs, and strong community bonds reduce social isolation, providing protective social scaffolding for diverse developmental trajectories.

### 13.4 Implications for global mental health

The mismatch model suggests that exporting Western diagnostic frameworks and interventions without cultural adaptation may be problematic. Instead, local communities should identify which traits cause genuine distress versus cultural nonconformity, ensuring that diagnostic criteria reflect local values and functional requirements rather than imposed Western norms. Interventions should build on existing cultural strengths, incorporating traditional practices and community resources that have historically supported neurodevelopmental diversity. Global mental health initiatives should address environmental factors such as pollution, inequality, and urbanization rather than focusing solely on individual treatment, recognizing that population-level neurodevelopmental outcomes reflect environmental conditions more than individual pathology.

## 14 Practical applications and systemic redesign

The Neurocognitive Mismatch Theory calls for a fundamental and comprehensive transformation of societal systems to better align with neurodevelopmental diversity. This section outlines practical applications and systemic redesigns across education, the workplace, urban planning, healthcare, and policy, demonstrating the coherence and feasibility of these proposed changes.

### 14.1 Educational transformation

Educational transformation from traditional classrooms to neurodiverse learning environments requires fundamental restructuring. Current systems typically enforce 6-h seated instruction with single-pace curriculum, expecting quiet, still behavior while prioritizing abstract learning and standardized testing. In contrast, redesigned systems would integrate multiple evidence-based modifications. Movement-integrated learning would incorporate standing desks, balance balls, and movement breaks every 20–30 min. Finland’s “Schools on the Move” program demonstrates the effectiveness of increased physical activity in schools (Haapala et al., 2021). Flexible pacing through self-paced modules would allow hyperfocus periods for autism profiles and topic-switching for ADHD profiles. Montessori education generally validates the effectiveness of self-paced, individualized learning (Lillard, 2012). Sensory accommodations including quiet zones, noise-canceling headphones, adjustable lighting, and textured materials address diverse sensory needs. Australian studies on sensory-based interventions in schools have reported a reduction in undesired behaviors (Mills and Chapparo, 2018). Project-based learning replaces abstract exercises with real-world applications. Project-based learning is widely recognized for improving engagement and critical thinking across diverse learners (Barron and Darling-Hammond, 2008).

### 14.2 Workplace restructuring

Workplace restructuring from traditional offices to cognitive diversity hubs requires comprehensive redesign. Current systems typically impose 9–5 fixed schedules with open office plans, constant meetings, multitasking demands, and heavy social networking emphasis. These environments systematically disadvantage neurodiverse individuals through sensory overload, rigid temporal structures, and communication barriers. Redesigned systems would implement flexible scheduling with core collaboration hours (such as 11 am-3 pm) while allowing flexible start and end times. Microsoft Japan’s 4-day work week exemplifies this approach, increasing productivity by 40% (Microsoft, 2019). Diverse workspace options would include private pods for deep focus suited to autism profiles, collaborative spaces for high-energy work matching ADHD needs, and outdoor working areas. Google’s campus design demonstrates this principle through meditation rooms, walking paths, and varied sensory environments. Task specialization would match cognitive profiles to job demands, placing detail-oriented autism profiles in roles requiring precision while utilizing ADHD profiles for crisis response and dynamic problem-solving. SAP’s Autism at Work program integrates people with autism into the company’s workforce (Pisano and Austin, 2016). Communication options would expand beyond verbal interaction to include written, visual, and asynchronous channels, reducing real-time social demands. GitLab’s all-remote model with documentation-first culture exemplifies how diverse communication styles can be supported effectively in professional environments.

### 14.3 Urban planning for neurodiversity

Urban planning transformation from traditional cities to sensory-conscious communities requires reimagining public spaces to support neurodevelopmental diversity. Green corridors consisting of nature paths connecting neighborhoods can significantly reduce sensory overload while providing restorative environments (Vukovic and Mingaleva, 2023). Quiet zones designated as low-stimulation areas in public spaces offer crucial sensory refuges for those overwhelmed by urban intensity, as demonstrated by London’s “Quiet London” campaign creating peaceful oases within the bustling city. Active transport infrastructure including extensive bike lanes and walking paths naturally supports movement needs while reducing reliance on sedentary transportation. Copenhagen’s cycling infrastructure provides a compelling model, correlating with better mental health outcomes across the population (Avila-Palencia et al., 2018). Community spaces such as maker spaces, gardens, and activity hubs provide diverse engagement options that accommodate different cognitive styles and sensory preferences, creating inclusive environments where various forms of participation and interaction are valued equally.

### 14.4 Healthcare system reform

Healthcare system reform from deficit models to support ecosystems fundamentally shifts the approach to neurodevelopmental differences. Environmental assessment becomes the first-line intervention, with clinicians evaluating sleep patterns, nutrition, movement opportunities, stress levels, and sensory environments before considering medication. This comprehensive evaluation recognizes that many symptoms attributed to inherent disorders may actually reflect environmental mismatches. Neuroplasticity interventions harness the brain’s adaptive capacity through cognitive training, mindfulness programs, and biofeedback. Cogmed working memory training exemplifies this approach, showing improvements in ADHD symptoms through targeted cognitive exercises (Klingberg, 2010). These interventions work by strengthening neural pathways rather than suppressing symptoms, offering lasting benefits without pharmaceutical side effects. A family systems approach replaces the traditional focus on individual “patients” by supporting entire family dynamics. This recognizes that neurodevelopmental traits affect and are affected by family interactions, stress levels, and communication patterns. Rather than isolating the identified patient, this approach strengthens the entire family’s capacity to support diverse neurodevelopmental profiles. Peer support networks connect families experiencing similar neurodevelopmental profiles, creating communities of mutual support and shared learning. These networks reduce isolation, provide practical strategies, and normalize neurodevelopmental diversity through lived experience sharing.

### 14.5 Policy recommendations

Policy recommendations for supporting neurodevelopmental diversity require comprehensive legislative and regulatory changes across multiple sectors. Education policy should mandate movement breaks, flexible seating options, and sensory accommodations in all schools, recognizing these as essential learning supports rather than special privileges. These requirements would establish minimum standards for cognitive accessibility comparable to existing physical accessibility mandates. Workplace legislation must extend disability accommodations to explicitly include cognitive diversity needs, moving beyond physical disabilities to recognize neurodevelopmental differences. This would legally protect flexible scheduling, sensory modifications, and communication accommodations as reasonable adjustments. Environmental regulations require stricter controls on neurotoxic pollutants known to impact neurodevelopment, alongside mandatory green space requirements in urban development projects. These measures would address environmental factors contributing to neurodevelopmental destabilization at the population level. Healthcare coverage reform should ensure insurance parity for environmental and behavioral interventions alongside medication, recognizing that non-pharmaceutical approaches often provide equal or superior long-term outcomes. This would make comprehensive environmental assessments, cognitive training programs, and family support services as accessible as stimulant prescriptions. Research funding priorities should shift toward studies examining environmental modifications rather than pharmaceutical development, redirecting resources toward understanding how to create supportive environments rather than suppressing symptoms. This reallocation would generate evidence for systemic interventions with population-level benefits rather than individual pharmaceutical management.

## 15 Alternative interventions and future research directions

### 15.1 Neuroplasticity-based interventions

This section details specific interventions that leverage the brain’s inherent capacity for neuroplasticity, its ability to adapt and change, providing evidence for their effectiveness in supporting neurodevelopmental function. The coherence of these approaches lies in their focus on strengthening neural pathways and addressing underlying mechanisms rather than merely suppressing symptoms.

#### 15.1.1 Cognitive training programs

Cognitive training programs targeting working memory enhancement have demonstrated significant therapeutic potential for neurodevelopmental conditions. Programs like Cogmed and CogniFit show measurable improvements in executive function through structured exercises that progressively challenge working memory capacity. Meta-analyses indicate significant effects on total ADHD and inattentive symptoms for reports by raters most proximal to the treatment setting, though these effects decreased substantially when outcomes were provided by probably blinded raters (Cortese et al., 2015). The key mechanisms involve strengthening prefrontal-parietal networks through repeated practice, essentially building neural highways through consistent use rather than relying on pharmaceutical augmentation. Attention training approaches offer complementary benefits through different mechanisms. Mindfulness-based interventions show promise for both ADHD and autism by teaching metacognitive awareness and emotional regulation skills. Eight-week MBSR (Mindfulness-Based Stress Reduction) programs demonstrate reduced ADHD symptoms and improved emotional regulation (Zylowska et al., 2008), providing tools for managing attention and reactivity without medication. Neurofeedback training represents a more direct approach, allowing individuals to modulate their own brain wave patterns through real-time feedback. This technique has shown significant improvement in behavior, attention, and IQ in pilot studies, and a significant treatment effect in meta-analyses, though better evidence from blinded assessments is required (Arns et al., 2020).

#### 15.1.2 Sensory integration approaches

Sensory integration approaches differ significantly between autism and ADHD profiles, reflecting their distinct neurodevelopmental characteristics. For autism profiles, sensory integration therapy directly addresses hyper-and hyposensitivities that can overwhelm daily functioning. Weighted blankets and compression vests provide proprioceptive input that reduces anxiety and improves focus by calming an overactive nervous system. Environmental modifications such as reducing fluorescent lighting and providing noise buffers create sensory-friendly spaces that prevent overload. Evidence suggests that Ayres Sensory Integration (ASI) intervention demonstrates positive outcomes for improving individually generated goals of functioning and participation for children with autism (Schaaf et al., 2018). For ADHD profiles, movement-based interventions address the fundamental need for physical activity and sensory input. Martial arts, dance, and swimming provide structured movement that channels hyperactivity while building executive function through complex motor sequences. Bilateral coordination exercises specifically target corpus callosum function, improving interhemispheric communication essential for attention regulation. Research suggests that time spent in nature can have a positive effect on attention (Kuo and Taylor, 2004). These findings underscore how matching interventions to specific neurodevelopmental profiles yields superior outcomes compared to one-size-fits-all approaches.

#### 15.1.3 Nutritional and metabolic interventions

Nutritional and metabolic interventions offer promising non-pharmaceutical approaches to supporting neurodevelopmental function. Dietary modifications demonstrate a notable impact. Elimination diets that remove artificial colors and preservatives have shown that restriction diets can reduce ADHD symptoms, with an estimated 8% of children with ADHD potentially having symptoms related to synthetic food colors (Nigg et al., 2012). This suggests that common food additives may exacerbate symptoms in susceptible individuals. Omega-3 supplementation addresses multiple pathways simultaneously, improving executive function while reducing systemic inflammation that can impact brain function. The gut-brain axis represents another intervention target, with probiotics and fermented foods modulating neurotransmitter production and immune function through microbiome optimization. For autism-related behaviors, ketogenic diets show promise (Lee et al., 2018). Metabolic support strategies target fundamental cellular processes underlying neurodevelopmental function. Mitochondrial support through CoQ10 and B-vitamins enhances cellular energy production, addressing the high metabolic demands of neural tissue. Methylation support via folate and B12 supplementation optimizes neurotransmitter synthesis pathways, potentially improving mood regulation and cognitive function. Anti-inflammatory protocols recognize the role of neuroinflammation in neurodevelopmental conditions, using targeted nutritional interventions to reduce inflammatory cascades that can impair neural function. These metabolic approaches work synergistically, supporting optimal brain function through multiple biochemical pathways rather than targeting single neurotransmitter systems as medications do.

#### 15.1.4 Technology-assisted interventions

Technology-assisted interventions leverage digital tools to provide personalized, adaptive support for neurodevelopmental differences. Virtual Reality (VR) applications offer unique therapeutic possibilities by creating controlled, customizable environments for skill development. For autism profiles, VR enables social skills training in environments where variables can be carefully managed, reducing overwhelming sensory input while practicing social interactions. ADHD focus training becomes engaging through gamified attention tasks that provide immediate feedback and reward, transforming concentration practice into an immersive experience. Sensory exposure therapy in graduated VR settings allows individuals to build tolerance to challenging stimuli at their own pace, offering a level of control impossible in real-world environments. Biometric monitoring represents another technological frontier, with wearable devices tracking arousal states and providing real-time feedback about physiological regulation. This technology helps individuals recognize early signs of dysregulation before reaching crisis points, enabling proactive self-management. Apps supporting emotional regulation through physiological awareness teach the connection between body states and emotions, building interoceptive awareness crucial for self-regulation. Sleep optimization technology addresses circadian disruptions common in both ADHD and autism, using light exposure tracking, movement monitoring, and environmental controls to support healthy sleep patterns. These technologies transform abstract concepts like “calm down” or “pay attention” into concrete, measurable skills with real-time guidance, making self-regulation strategies more accessible and effective.

### 15.2 Future research directions and testable hypotheses

The Neurocognitive Mismatch Theory generates several specific predictions that can be empirically tested through rigorous research designs. These future research directions are crucial for validating the theory and translating its principles into actionable interventions.

#### 15.2.1 Longitudinal cohort studies

Hypothesis 1 proposes that children in environments with greater nature access, movement opportunities, and reduced academic pressure will show lower rates of ADHD/autism-related impairment over time. This hypothesis directly tests whether environmental factors, rather than inherent neurological differences, drive functional impairment. The proposed study would conduct a 10-year longitudinal comparison of neurodevelopmental outcomes across contrasting environments. Traditional urban schools would be compared with forest schools to examine the impact of nature immersion and movement-based learning. High-pressure academic systems would be contrasted with play-based learning environments to assess how performance demands affect neurodevelopmental trajectories. Communities with varying levels of green space and air quality would be studied to determine dose–response relationships between environmental quality and neurodevelopmental outcomes. This comprehensive design would track the same children over time, measuring not just diagnostic rates but functional outcomes, quality of life, and adaptive skills. By comparing environments that differ systematically in their alignment with evolved developmental needs, this research could provide strong evidence for or against the environmental mismatch hypothesis.

#### 15.2.2 Intervention trials

Hypothesis 2 directly challenges the primacy of pharmaceutical intervention by proposing that environmental modifications will produce comparable or superior outcomes to medication without adverse effects. This hypothesis tests whether addressing environmental mismatch can match or exceed the benefits of symptom suppression through medication. The proposed randomized controlled trial would compare four conditions: stimulant medication alone, comprehensive environmental modification (incorporating structured movement opportunities, nutritional optimization, and sensory accommodations), a combined approach using both strategies, and a control group receiving standard care. This design allows for direct comparison of pharmaceutical versus environmental interventions while also testing potential synergistic effects. Outcome measures would extend beyond symptom reduction to include executive function assessments, quality of life indicators, and long-term academic and occupational success. By tracking participants over multiple years, the study could capture not only immediate effects but also sustainability of improvements and potential adverse effects of each approach. This research design addresses a critical gap in current literature, which rarely compares comprehensive environmental interventions against medication in head-to-head trials, potentially revolutionizing treatment approaches if environmental modifications prove equally effective without the risks associated with long-term pharmaceutical use.

#### 15.2.3 Epigenetic research

Hypothesis 3 addresses the biological mechanisms underlying environmental effects by proposing that environmental stressors produce measurable epigenetic changes associated with ADHD/autism traits that are reversible through intervention. This hypothesis bridges the gap between environmental factors and biological outcomes, potentially explaining how external conditions translate into neurodevelopmental differences. The proposed study would collect DNA methylation profiles from children before and after environmental interventions, providing direct evidence of epigenetic plasticity in response to environmental change. By comparing epigenetic markers in high-stress versus low-stress environments, researchers could identify specific methylation patterns associated with neurodevelopmental destabilization. Tracking intergenerational transmission patterns in families migrating between environments would reveal whether epigenetic changes induced by stressful conditions can be reversed when families move to more supportive environments, and whether these reversals affect offspring. This design uniquely captures both the dynamic nature of epigenetic regulation and its potential for intergenerational transmission. Such research could demonstrate that neurodevelopmental traits are not fixed genetic destinies but rather plastic responses to environmental conditions, with profound implications for intervention timing and approach. If environmental improvements can reverse stress-induced epigenetic changes, this would provide biological validation for systemic rather than pharmaceutical interventions.

#### 15.2.4 Cross-cultural natural experiments

Hypothesis 4 examines cultural variation by proposing that societies with specific cultural practices such as extended families, nature integration, and movement-based learning will show different neurodevelopmental trajectories. This hypothesis tests whether cultural factors that align with evolved developmental needs protect against neurodevelopmental destabilization. The proposed study would conduct comparative prevalence and impairment studies across cultures, moving beyond simple diagnostic rates to examine functional outcomes in different cultural contexts. This would reveal whether traits labeled as disorders in Western contexts cause less impairment in societies with different social structures and expectations. Migration studies examining changes in neurodevelopmental outcomes would provide natural experiments, tracking how children’s functioning changes when families move between cultures with different practices. For instance, comparing children from traditional societies before and after migration to urban Western environments could reveal how cultural transition affects neurodevelopmental expression. Policy evaluation as countries adopt different educational and healthcare approaches would capture real-time effects of systemic changes. As nations implement movement-based learning or reduce academic pressure, researchers could track population-level changes in neurodevelopmental outcomes. This comprehensive approach would demonstrate whether neurodevelopmental “disorders” are universal biological conditions or culturally-mediated expressions of mismatch between evolved traits and local environments.

#### 15.2.5 Biomarker development

Hypothesis 5 proposes that objective biomarkers can distinguish between inherent neurodiversity and environmental destabilization, addressing a fundamental question about the nature of neurodevelopmental conditions. This hypothesis suggests that different biological signatures characterize stable neurodevelopmental variations versus environmentally-induced dysfunction. The proposed research would develop comprehensive biomarker panels combining genetic, epigenetic, inflammatory, and neuroimaging markers to create multidimensional biological profiles. Genetic markers would identify inherited neurodevelopmental variations, while epigenetic patterns would reveal environmental influences on gene expression. Inflammatory markers could indicate ongoing environmental stress responses, and neuroimaging would show structural and functional brain differences. By integrating these diverse biological measures, researchers could potentially differentiate between individuals whose traits represent stable neurodiversity and those experiencing environmental destabilization of otherwise functional variations. Validating markers that predict intervention response would provide clinical utility beyond diagnosis. If certain biomarker profiles predict better response to environmental modifications versus medication, this would enable precision medicine approaches to neurodevelopmental support. Creating personalized intervention algorithms based on biomarker profiles would move beyond one-size-fits-all treatments to match specific interventions with individual biological patterns. This research could revolutionize how we conceptualize and treat neurodevelopmental differences, shifting from categorical diagnoses to dimensional understanding of person-environment fit, ultimately enabling more effective, personalized support strategies.

#### 15.2.6 Systems-level interventions

Hypothesis 6 represents the most ambitious test of the theory by proposing that community-wide environmental changes will reduce population-level neurodevelopmental impairments. This hypothesis moves beyond individual interventions to examine whether systemic environmental modifications can shift entire population distributions of neurodevelopmental outcomes. The proposed natural experiments would evaluate cities implementing comprehensive neurodiversity-friendly policies, tracking changes in diagnosis rates, functional outcomes, and quality of life metrics across entire urban populations. These evaluations would examine cities that integrate green spaces, reduce sensory pollution, mandate movement breaks in schools, and create quiet zones in public spaces. By comparing similar cities with and without such policies, researchers could establish whether environmental design at scale affects neurodevelopmental outcomes at the population level. Studying companies adopting cognitive diversity employment practices would reveal whether workplace accommodations reduce impairment in adult populations. Tracking employee wellness, productivity, and retention in companies with flexible scheduling, diverse workspace options, and task-matching programs compared to traditional employers would demonstrate whether occupational environments can support or undermine neurodevelopmental functioning. Assessing schools transforming to movement-based, flexible learning models would provide critical data on whether educational reform can prevent the emergence of impairment. Comparing academic, social, and emotional outcomes between traditional and transformed schools would test whether aligning educational environments with developmental needs reduces the need for diagnosis and treatment. These research directions would provide empirical validation or refutation of the Neurocognitive Mismatch Theory while generating practical insights for supporting neurodevelopmental diversity. If population-level interventions prove effective, this would fundamentally shift intervention focus from treating individuals to creating supportive environments for all.

## 16 Discussion

This paper has proposed a theoretical model, Neurocognitive Mismatch Theory, to explain how neurodevelopmental traits associated with ADHD and autism may become destabilized within post-agricultural, market-based societies. This framework is hypothesis-generating, not empirically confirmed. While it draws on evidence from developmental neuroscience, evolutionary theory, environmental health, and social epidemiology, the central claim remains correlational: dysfunction arises when inherently functional traits are forced into incompatible environments. One limitation of the model is the challenge of disentangling causality from correlation. Although research supports associations between environmental stressors and neurodevelopmental instability (Lupien et al., 2009; Luby et al., 2013), direct causal mechanisms are difficult to establish in human populations. Ethical constraints prevent controlled experiments on early-life adversity or prenatal stress. Additionally, most epigenetic evidence is derived from animal models (Rodgers et al., 2013; Bohacek and Mansuy, 2015), which, while informative, limit generalizability. Another constraint is cultural specificity. This paper focuses primarily on Western, industrialized societies, where bureaucratic schooling, screen exposure, and socioeconomic inequality are pervasive. Cross-cultural studies suggest that attentional variability and sensory sensitivity may be less pathologized in non-industrial settings (Henrich et al., 2010). The mismatch model may therefore reflect specific institutional contexts rather than universal dynamics. Despite these limitations, the framework contributes to a growing body of literature questioning the deficit-based medicalization of neurodevelopmental variation. It aligns with neurodiversity paradigms that view ADHD and autism not as disorders, but as part of the natural spectrum of human cognitive ecology (Walker, 2021). It also engages with philosophical critiques of psychiatry that emphasize the sociopolitical construction of disorder categories (Illich, 1976; Whitaker, 2010). The model generates testable predictions. For instance, environments with lower sensory load, reduced behavioral conformity, or decreased psychosocial stress should produce better outcomes in individuals with ADHD or ASD profiles; without pharmacological intervention. Similarly, societies with less rigid productivity demands may exhibit lower prevalence or severity of dysfunction. These hypotheses can be explored through comparative cultural studies, longitudinal epigenetic research, and policy-level experimentation in educational and workplace design. Finally, the theory has ethical implications. It suggests that treatment should begin not with the individual, but with the structure. If dysfunction is emergent rather than intrinsic, then systems must adapt to human diversity; not the other way around. Medication may have a role in specific cases, but as a last resort; not a default solution to systemic inflexibility (Cortese et al., 2015; Volkow et al., 2009). Re-centering intervention on context, not correction, aligns not only with emerging science, but with a more humane approach to neurodevelopment. The traits associated with ADHD and autism are not defects of the brain but divergences in neurodevelopment that become destabilized under incompatible environmental conditions. This paper has argued that dysfunction arises not from the inherent traits themselves, but from their interaction with modern systems that demand uniformity, overstimulation, and behavioral conformity. Cognitive diversity is not a problem to be fixed, but a reality to be accommodated. When institutions are designed around narrow norms of attention, behavior, and productivity, they generate disorder where there might otherwise be difference. The pathology lies in the structure; not the person. Reframing neurodevelopmental conditions through the lens of mismatch opens new pathways for understanding, support, and inclusion. It shifts the ethical responsibility from the individual to the environment and challenges us to imagine systems where divergence is not merely tolerated, but valued.
